# Fibroblast Growth Factor Receptor 2 (FGFR2) Is Required for Corneal Epithelial Cell Proliferation and Differentiation during Embryonic Development

**DOI:** 10.1371/journal.pone.0117089

**Published:** 2015-01-23

**Authors:** Jinglin Zhang, Dinesh Upadhya, Lin Lu, Lixing W. Reneker

**Affiliations:** 1 State Key Laboratory of Ophthalmology, Zhongshan Ophthalmic Center, Sun Yat-Sen University, Guangzhou, China; 2 Dept. of Ophthalmology, Mason Eye Institute, University of Missouri, Columbia, Missouri, United States of America; University of Reading, UNITED KINGDOM

## Abstract

Fibroblast growth factors (FGFs) play important roles in many aspects of embryonic development. During eye development, the lens and corneal epithelium are derived from the same surface ectodermal tissue. FGF receptor (FGFR)-signaling is essential for lens cell differentiation and survival, but its role in corneal development has not been fully investigated. In this study, we examined the corneal defects in F*gfr2* conditional knockout mice in which Cre expression is activated at lens induction stage by Pax6 P0 promoter. The cornea in *LeCre, Fgfr2^loxP/loxP^* mice (referred as *Fgfr2^CKO^*) was analyzed to assess changes in cell proliferation, differentiation and survival. We found that *Fgfr2^CKO^* cornea was much thinner in epithelial and stromal layer when compared to *WT* cornea. At embryonic day 12.5–13.5 (E12.5–13.5) shortly after the lens vesicle detaches from the overlying surface ectoderm, cell proliferation (judged by labeling indices of Ki-67, BrdU and phospho-histone H3) was significantly reduced in corneal epithelium in *Fgfr2^CKO^* mice. At later stage, cell differentiation markers for corneal epithelium and underlying stromal mesenchyme, keratin-12 and keratocan respectively, were not expressed in *Fgfr2^CKO^* cornea. Furthermore, Pax6, a transcription factor essential for eye development, was not present in the *Fgfr2^CKO^* mutant corneal epithelial at E16.5 but was expressed normally at E12.5, suggesting that FGFR2-signaling is required for maintaining Pax6 expression in this tissue. Interestingly, the role of FGFR2 in corneal epithelial development is independent of ERK1/2-signaling. In contrast to the lens, FGFR2 is not required for cell survival in cornea. This study demonstrates for the first time that FGFR2 plays an essential role in controlling cell proliferation and differentiation, and maintaining Pax6 levels in corneal epithelium via ERK-independent pathways during embryonic development.

## Introduction

The cornea is a transparent tissue on the surface of the eye with refractive properties for bending light rays. The development of the vertebrate cornea involves inductive interactions between surface ectodermal and mesenchymal tissues [1]. At embryonic day 8.5 to 9.0 (E8.5–9.0), a thickened region of the head ectoderm, defined as the lens placode, gives rise to both the lens and the presumptive corneal epithelium. The primitive corneal epithelium forms after the lens vesicle detaches from the overlying surface ectoderm. At around E12.0–12.5, the perioptic mesenchyme (mostly neural crest cells) migrates into the space between the lens and the primitive corneal epithelium [1,2]. At E14.5–15.5 in the mouse eye, the posterior mesenchymal cells closest to the lens differentiate into a thin layer of corneal endothelium, and the anterior chamber subsequently forms between the lens and cornea. The mesenchymal cells between the corneal epithelium and endothelium begin to differentiate into keratocytes and form corneal stroma. The corneal epithelium continues to differentiate after birth and, upon eyelid opening at two weeks of age, the corneal epithelium expands from two cell layers to a self-renewing, stratified epithelium comprising eight to 10 cell layers [3,4]. The fully developed cornea is composed of three layers derived from two embryonic germ tissues: a stratified corneal epithelium with surface ectoderm origin on the outer surface, expressing the keratin 3 and 12 (K3/K12) pair [5]; the stromal layer underneath, sparsely populated by keratocytes composed of highly aligned collagen, and the inner surface of the cornea, covered by a single-layer endothelium.

Corneal injury and disease can lead to opacification, neovascularization, fibrosis and defective wound healing. These pathological conditions together constitute the second leading cause of blindness worldwide [6]. Understanding the inductive factors and signals that regulate corneal cell proliferation and differentiation has important implications for the development of therapeutic approaches for controlling corneal repair and homeostasis and preventing blindness. Several lines of evidence support the integral role of fibroblast growth factors (FGFs) in corneal cell proliferation and differentiation [7]. As many as 22 FGFs have been identified in vertebrates [8]. FGF signaling is activated through binding of the growth factor to its cell surface receptors to stimulate receptor dimerization and activation of receptor tyrosine kinases, ultimately leading to activation of various downstream signal transduction cascades [9]. Four fibroblast growth factor receptor (FGFR) genes (*FGFR1* to *FGFR4*) have been cloned and identified in mammals. Additionally, multiple FGFR isoforms, differing in structure and ligand affinity, can be generated through alternative splicing of primary transcripts. For example, two FGFR2 variants, FGFR2IIIb and FGFR2IIIc, are generated by alternative splicing at the second half of Ig domain III of the *FGFR2* locus [10,11]. During corneal development, FGF-7 and FGF-10 are secreted by corneal mesenchymal cells and both can bind with affinity to FGF receptor 2 (FGFR2-IIIb) isoform, which is expressed mainly in limbal and central corneal epithelium [12–14]. These expression patterns imply that FGFR2-signaling may promote limbal stem cell proliferation and participate in modulation of corneal epithelium renewal and homeostasis. In vitro functional studies have shown that FGF-7 enhances the growth and proliferation of cultured corneal epithelial cells but does not significantly affect motility [15] [16]. Topical application of FGF-7 was shown in vivo and in vitro to accelerate corneal epithelial wound healing [17–19]. In an investigation of the role of FGFR activation in corneal development, transgenic mice overexpressing FGF-7 or FGF-10 in the developing lens (starting as early as E11.5) exhibited hyperproliferative corneal epithelial cells that subsequently were induced to alter their cell fate from corneal epithelium to lacrimal gland epithelium [20–22]. In another study of transgenic mice, overexpression of FGF-3, another member in the FGF family also capable of activating FGFR2IIIb, was found to stimulate epithelial-to-glandular transformation in the developing cornea of the transgenic mice [23]. However, when excess FGF-7 was induced in the corneal epithelium of young mice, the main phenotype was hyperplasia in the epithelial layer, without alteration in cell fate [24]. The corneal epithelium increased in thickness from 6 or 7 cell layers to more than 20 cell layers, with extended K14 expression from the basal to suprabasal to superficial layers. Phenotypic variations caused by excessive FGF-7 were found in the eyes of embryos and young pups, which may be explained by the age-dependent differences of FGFR2-activated signaling network in developing corneal epithelium and the plasticity of progenitor cells. However, these gain-of-function studies have not defined the normal biological role of FGFR2 in corneal development.

The function of FGFR2 in the development of ocular surface ectodermal tissues, including the lens and the lacrimal glands, has been investigated using the *Fgfr2* conditional knockout mice (referred as *Fgfr2* ^*CKO*^) driven by a surface ectodermal Cre line, the *Le-Cre* [25–27]. These studies revealed that the FGFR2-activated Ras-ERK signaling pathway is essential for cell survival and cell cycle exit during ocular lens development and for induction of the lacrimal glands. Although FGFR2 is known to be expressed in the corneal epithelium, the developmental changes in the cornea of *Fgfr2* conditional knockout mice have not been investigated in detail. In this study, we demonstrate that FGFR2 is required for corneal epithelial cell proliferation at the stage shortly after the lens vesicle detaches from the surface ectoderm. In contrast to its role in the lens, FGFR2 is not essential for corneal epithelial cell survival. Furthermore, we demonstrate that FGFR2 plays an essential role in maintaining the Pax6 expression, independent of ERK-signaling, in corneal epithelium. In the absence of Pax6, differentiation and maturation of corneal epithelium is inhibited in *Fgfr2* ^*CKO*^ mice. We also found that the abnormal development of corneal epithelium in *Fgfr2* ^*CKO*^ mice does not affect the migration and proliferation of the corneal mesenchymal cells, but prevents these cells from differentiating into mature keratocytes, suggesting that loss of FGFR2 interferes with the signaling interactions between the corneal epithelium and underlying mesenchyme.

## Materials and Methods

### Mice

Mice carrying the *Fgfr2* flox alleles and the *Le-Cre* transgenic mice were obtained from Dr. Michael Robinson (Miami University, Oxford, OH, USA), with permission from Drs. David Ornitz and Ruth Ashery-Padan, respectively [25,27,28]. The ERK1/2 double deletion mice (*LeCre-Mapk1* ^*fl/f*^ *l;Mapk3* ^*-/-*^) were described previously [29]. To generate *Fgfr2* conditional knockout mice, *Le-Cre* mice were bred to *Fgfr2* ^*flox/flox*^ mice and the heterozygous offspring *Le-Cre;Fgfr2* ^*flox/+*^ mice were then crossed with *Fgfr2* ^*flox/flox*^ to make *Le-Cre;Fgfr2* ^*flox/flox*^ (referred *as Fgfr2* ^*CKO*^) mice. The *Le-Cre* mice in all experiments were heterozygous for the transgene. The *Fgfr2* ^*loxP/loxP*^ mice are referred to as *wild type* (*WT*). Because *Le-Cre* hemizygous transgenic mice were shown to develop eye abnormalities on some genetic backgrounds [30], we recently crossed the *Fgfr2* ^*CKO*^ mice with C57BL6J mice to examine potential defects in the cornea of *Le-Cre;Fgfr2* ^*flox/+*^ mice. Animal use was in accordance with the Association of Research in Vision and Ophthalmology (ARVO) Statement for the Use of Animals in Ophthalmic and Vision Research, and all experimental procedures were approved by the Animal Care and Use Committee of the University of Missouri-Columbia.

### Histology, immunohistochemistry and immunofluorescence

Embryonic and newborn mouse heads were fixed in 4% paraformaldehyde for 2 hours or overnight and processed for histological analysis by hematoxylin and eosin (H&E) staining, as described previously [31]. For immunohistochemistry and immunofluorescence, the following primary antibodies were used: anti-Ki67 (M7249, Dako, Carpinteria, CA, USA); anti-keratin-14 (K14) (PBR-159P) and anti-Pax6 (PBR-278P), both from Covance Inc, Princeton, NJ, USA; anti-N-cadherin (33–3900, Zymed, Camarillo, CA, USA), and anti-phospho-histone H3 (sc-8656-R, Santa Cruz Biotechnology, Santa Cruz, CA, USA). Anti-keratocan and anti-keratin-12 (K12) antibodies were gifts from Dr. Chia-yang Liu at University of Cincinnati (Cincinnati, OH, USA) [24]. For immunofluorescence, Alexa Fluor conjugated secondary antibodies were purchased from Invitrogen (Carlsbad, CA, USA) and cell nuclei were counterstained with 4′,6-diamidino-2-phenylindole (DAPI). The signal-enhancement TSA kit (NEL741B001KT, PerkinElmer, Boston, MA, USA) was used for immunofluorescence against Ki67, K12 and N-cadherin. For immunohistochemistry, biotinylated secondary antibodies were from Vector Laboratories (Burlingame, CA, USA) and color was developed by using 3, 3′-diaminobenzidine as a substrate (D4293, Sigma, St Louis, MO, USA). Sections were counterstained for cell nuclei by hematoxylin.

### Brdu incorporation and TUNEL assays

5-Bromo-2′-deoxyuridine (BrdU) was administered intraperitoneally into pregnant mice at a concentration of 0.1μg/gm body weight and labeled for 1 hour prior to embryo isolation. BrdU immunohistochemistry was performed as previously described [32]. Terminal deoxynucleotidyl transferse dUTP nick end labeling (TUNEL) assay was performed with in situ apoptosis detection kit (S7165, Millipore, Billerica, MA, USA), following the manufacturer’s instructions.

### Statistical analysis

Quantification of cell proliferation was performed by determining the fraction of labeled nuclei over the total number of nuclei present in a given section. A minimum of 3 embryos for each genotype were analyzed at a given time point. Data are expressed as mean ± SEM and p-values were calculated using Mann-Whitney Test (p<0.05 was considered significant).

## Results

### Conditional deletion of *Fgfr2* in ocular surface ectoderm affects corneal development

**Fig 1:**
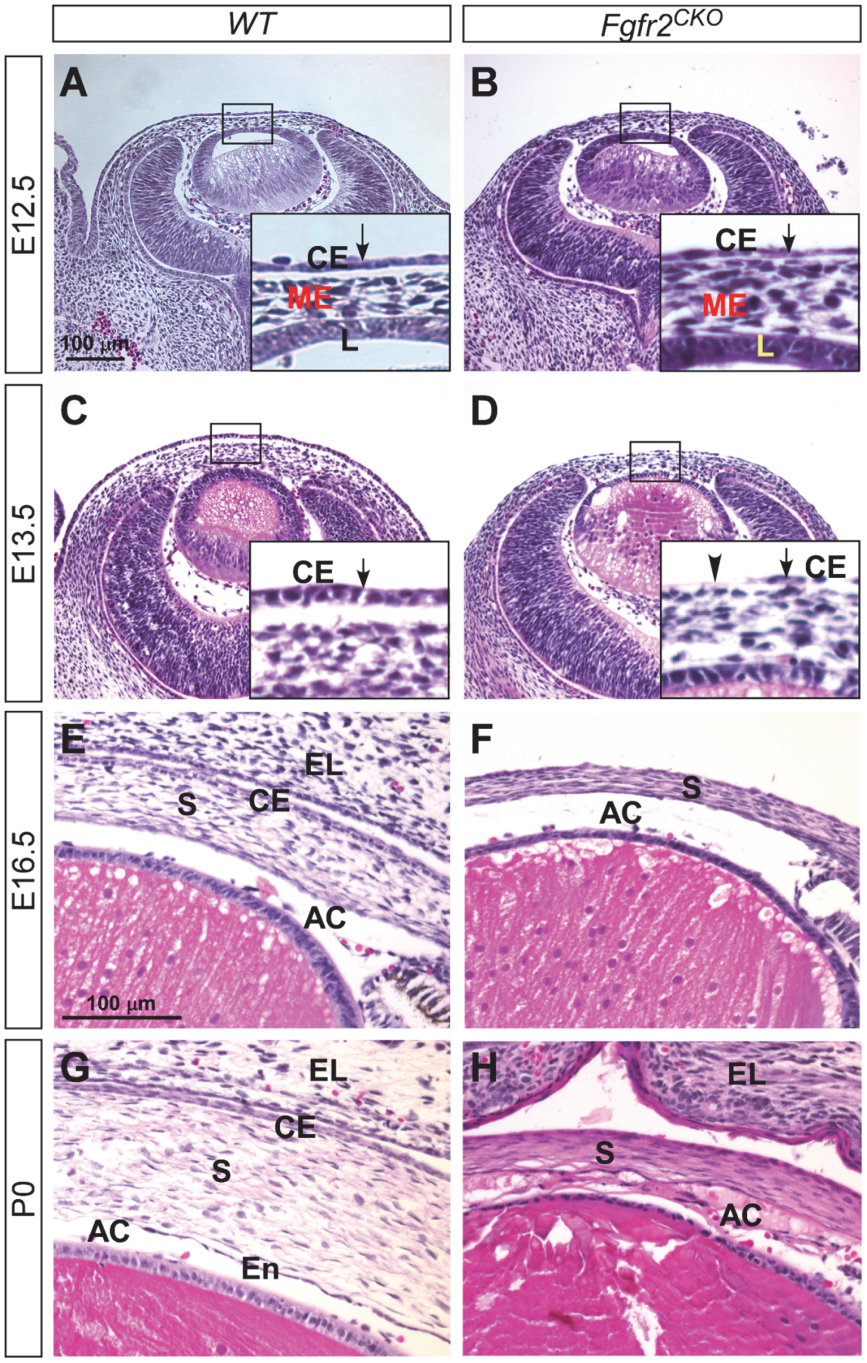
Corneal development (H&E staining) in *WT* and *Fgfr2* ^CKO^ eyes. A, B) At E12.5, ocular mesenchymal cells migrated into the space between the lens (L) and the corneal epithelium (CE, arrow in enlarged inset) in both *WT* and *Fgfr2* ^*CKO*^ eyes. The corneal epithelial layer in *Fgfr2* ^*CKO*^ eyes was slightly thinner than that in *WT* eyes. C, D) At E13.5, the corneal epithelial layer was significantly thinner in *Fgfr2* ^*CKO*^ eyes when compared to *WT* eyes. In some areas, the epithelial cells were absent (arrowhead in D, insert). E, F) At E16.5, corneal stroma (S) was thinner but cells were more densely packed and eosin-staining was intensified in *Fgfr2* ^*CKO*^ cornea as compared to *WT*. Anterior chamber (AC) was formed in both genotypes. As previously reported, eyelid (EL) fusion did not occur in *Fgfr2* ^*CKO*^ eyes. G, H) At P0, *Fgfr2* ^*CKO*^ mice developed additional defects in the anterior segments, including abnormal accumulation of extra cells and tissues in the anterior chamber (AC), and loss of a distinctive corneal endothelial (En) layer.

In eye development, the surface ectoderm gives rise to both corneal epithelium and the lens. The essential role of FGFR2 in lens development has been shown in the *Fgfr2* conditional deletion mice by a surface ectoderm driver *Le-Cre* [25]. However, the function of FGFR2 in corneal epithelial development has not been assessed. We first examined corneal development in the eyes of *Fgfr2* ^*CKO*^ mice by histology (H&E staining). We found that at E12.5 the presumptive corneal epithelium in *Fgfr2* ^*CKO*^ eyes looked either similar to or slightly thinner than the corneal epithelium in *WT* control eyes (Fig. 1A and 1B). In both genotypes, the ocular mesenchymal cells had migrated into the space between the lens and the corneal epithelial layer. At E13.5, however, developmental abnormalities were noted in *Fgfr2* ^*CKO*^ corneas (Fig. 1C and 1D). The corneal epithelial layer in *Fgfr2* ^*CKO*^ eyes was significantly thinner than normal. The cell density in corneal epithelium was reduced and cells were absent in some areas (indicated by the arrowhead in Fig. 1D). The corneal defects in *Fgfr2* ^*CKO*^ eyes progressed beyond the epithelial layer. The *WT* corneal epithelium consisted of basal cuboidal and superficial flattened cells, whereas the *Fgfr2* ^*CKO*^ corneal epithelium consisted entirely of flattened cells. In E16.5 *Fgfr2* ^*CKO*^ eyes, corneal stroma was thinner but cells were more densely packed, with intensified eosin staining, when compared to the age-matched *WT* corneal stroma (Fig. 1E and 1F). The anterior chamber was formed in both genotypes (Fig. 1E and 1F). As previously reported, eyelid fusion did not occur in *FGFR2* ^*CKO*^ eyes [33,34]. Additional defects also occurred in anterior segments development of *Fgfr2* ^*CKO*^ eyes, including abnormal accumulation of extra cells and tissues in the anterior chamber and a defective corneal endothelial layer (Fig. 1G and 1H). These data suggest that in addition to normal lens development [25], FGFR2 function is also required for normal corneal development.

### FGFR2 is not required for corneal epithelial cell survival

**Fig 2:**
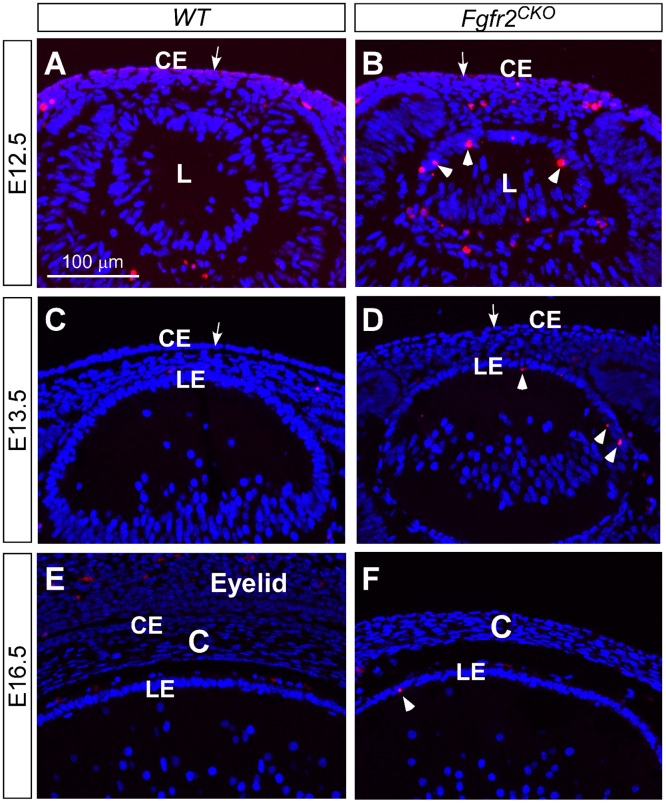
Apoptosis detected by TUNEL assay. Loss of FGFR2-induced apoptosis in mouse lens (l) (arrowheads in B, D, and F), but not in cornea epithelium (CE) (arrows in A-D). In E12.5 *WT* and *Fgfr2* ^*CKO*^ eyes, TUNEL-positive cells were found in the corneal mesenchymal cells and in the hyaloid cells of the primary vitreous. (LE, lens epithelium; C, cornea)

The lens has been shown to undergo the loss of FGFR2-induced apoptosis during development [25]. To investigate whether the reduced cell density in corneal epithelium of FGFR2CKO mice is caused by apoptosis, TUNEL assay was performed on eyes at different ages (Fig. 2A through 2F). We found a drastic increase in apoptotic cells in Fgfr2CKO lenses, a result consistent with the previous finding [25]. The corneal epithelial layer in both WT and Fgfr2CKO eyes, however, did not demonstrate an increase in apoptotic cells, suggesting that FGFR2 is not required for corneal epithelial cell survival. TUNEL-positive cells were also detected in other ocular tissues, including the corneal mesenchymal cells and the hyaloid vascular cells in both WT and Fgfr2CKO eyes, but no significant difference was noted between the two genotypes.

### FGFR2 is essential for corneal epithelial cell proliferation during early-stage development

**Fig 3:**
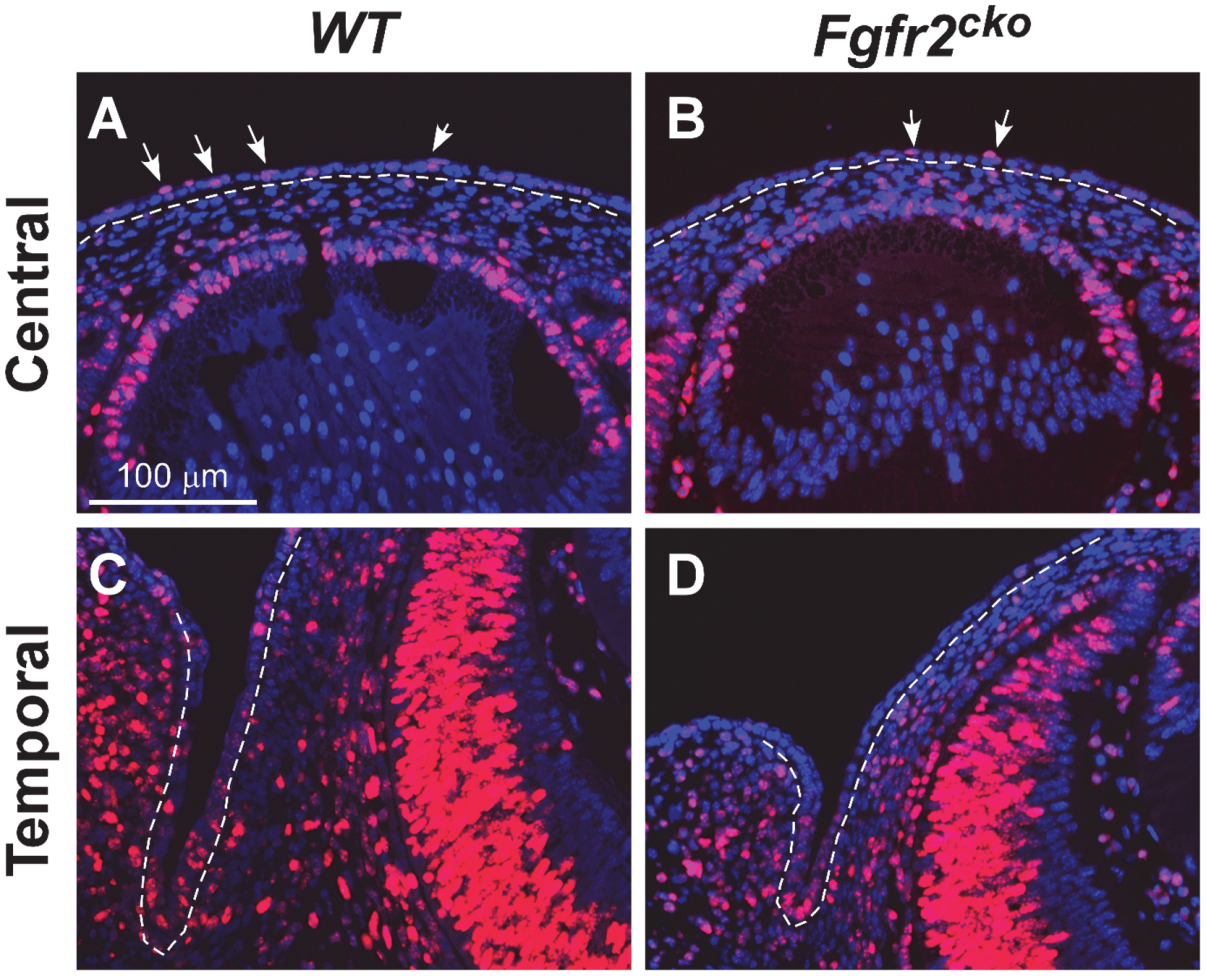
Ki67 immunofluorescence in the central and temporal areas of E13.5 *WT* and *Fgfr2* ^CKO^ eyes. Compared to Ki67-expressing cells *WT* eyes (arrows in A, C), there was a significant reduction in Ki67-expressing cells in the surface ectodermal layer of *Fgfr2* ^*CKO*^ eyes (arrows in B, D). The central epithelial layer later forms the corneal epithelium, and peripheral cells differentiate into limbal and conjuctival epithelium. There was no obvious difference between the *WT* and *Ffgr2* ^*CKO*^ mice in the number of Ki67-positive cells in the stroma and lens.

We investigated whether the reduced cell number in *Fgfr2* ^*CKO*^ corneal epithelium was affected by defects in cell proliferation. Immunofluorescence of Ki67, a cell proliferation marker expressed in active cycling cells, was performed on E13.5 eyes, the age when the corneal epithelial defect becomes apparent. We found that Ki67-positive cells were markedly reduced in both the central and peripheral regions of the corneal epithelial layer in *Fgfr2* ^*CKO*^ eyes when compared to the littermate *WT* eyes (Fig. 3A through 3D). In contrast to the differences between *Fgfr2* ^*CKO*^ and *WT* Ki67-positive cells in corneal epithelium, no noticeable difference was found between the two genotypes in the number of Ki67-positive cells in the lens epithelium and corneal stroma.

**Fig 4:**
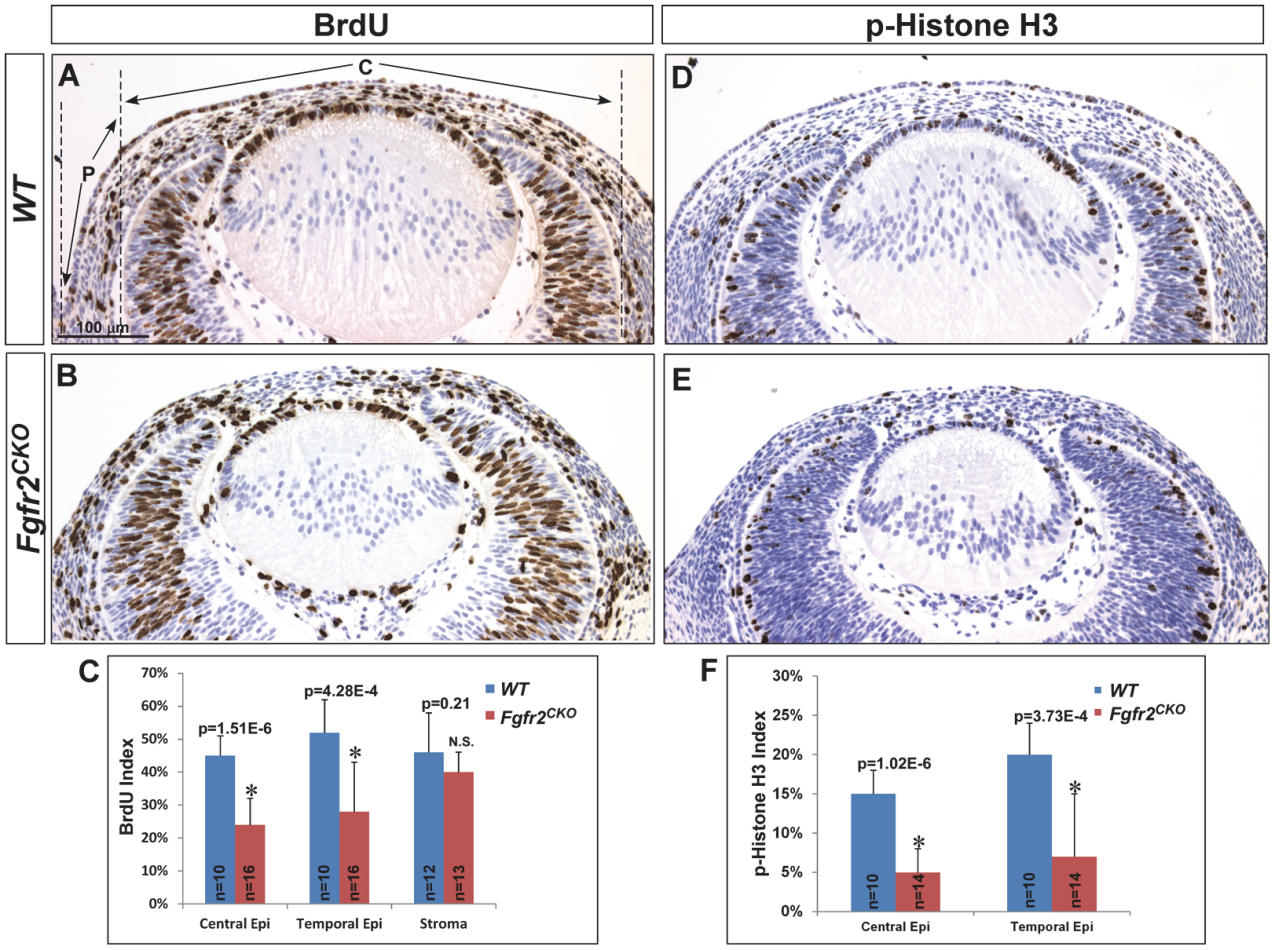
Cell proliferation analysis. A, B, C) BrdU incorporation assay in E13.5 *WT* and *Fgfr2* ^*CKO*^ mice demonstrated that BrdU-positive cells were significantly reduced in the corneal epithelium of *Fgfr2* ^*CKO*^ mice as compared to *WT* mice. The surface epithelium is divided into central (C) and peripheral (P) area as illustrated in A. Quantitative analysis confirmed that BrdU-labeling index was decreased in both regions in *FGFR2* ^*CKO*^ eyes. Note: only the data from temporal side is shown in C. In contrast to corneal epithelium, BrdU indices in corneal stroma were not significantly different between the two genotypes. D, E, F) Immunohistochemistry of phospho-histone H3 (p-H3) in E13.5 eyes. Similar to the BrdU results, p-H3-labeling indices were reduced in both the central and peripheral corneal epithelial layer in *Fgfr2* ^*CKO*^ mice. (n = sections counted; *p<0.0005; N.S. = not significant)

To further analyze the effect of FGFR2 deficiency on cell cycle progression, BrdU incorporation assay and expression of phospho-histone H3 (p-H3) were performed on E13.5 eyes (Fig. 4A through 4F). BrdU is a marker for cells in the S-phase, whereas p-H3 is a marker for G2-M phase cells. Consistent with the results of Ki67 expression, both BrdU and p-H3–labeled cells were significantly reduced in the corneal epithelial layer of E13.5 *Fgfr2* ^*CKO*^ eyes when compared to *WT* eyes. To quantify the labeling index, the ocular surface ectodermal layer was divided into central and peripheral areas, as illustrated in Fig. 4A. In the central area, *WT* BrdU and p-H3 indices were 0.45±0.06 and 0.15±0.03, respectively, whereas they were 0.24±0.08 and 0.05±0.03, respectively, in *Fgfr2* ^*CKO*^ cornea (p<0.05), a statistically significant decrease for both cell cycle markers when compared to control *WT* eyes. For the peripheral area, BrdU and p-H3 indices were obtained from the temporal side where lacrimal gland budding occurs at the fornix. Similar to the central area, indices were significantly decreased in *Fgfr2* ^*CKO*^ eyes (0.28±0.15 for BrdU and 0.07±0.07 for p-H3 in *Fgfr2* ^*CKO*^ eyes vs. 0.52±0.1 and 0.20±0.04, respectively, in *WT* eyes). BrdU index in the central area was calculated to determine whether *Fgfr2* deletion in surface ectodermal tissues affects cell proliferation in corneal mesenchyme migrated (Fig. 4C). There was no statistical difference between *WT* and *FGFR2* ^*CKO*^ mice (0.46±0.12 vs. 0.40±0.06), suggesting that cell proliferation in corneal stromal cells is not affected by deletion of *Fgfr2* in corneal epithelium. Taken together, our data suggest that after lens vesicle detachment, FGFR2 is required for cell proliferation in the overlying surface ectoderm, which later differentiates into the corneal epithelium, lacrimal glands and conjunctival epithelium.

### FGFR2 is important for differentiation of corneal epithelial cells and for maintaining Pax6 expression in these cells

**Fig 5:**
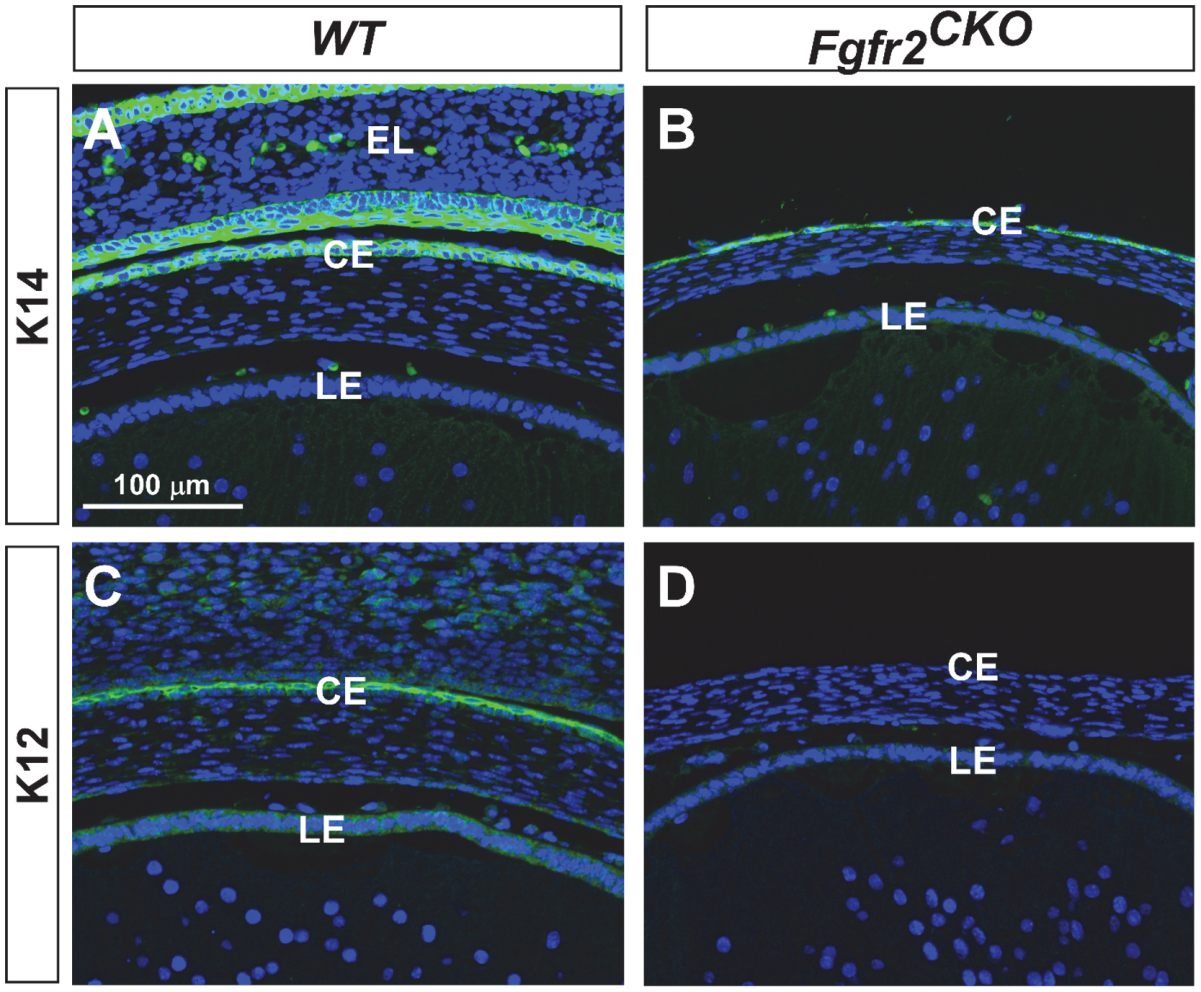
Corneal epithelial cell differentiation. A, B) K14 was expressed in corneal epithelium (CE) in both *WT* and *Fgfr2* ^*CKO*^ eyes, and in eyelid (EL) epithelia in *WT* eyes. Eyelid fusion did not occur in *Fgfr2* ^*CKO*^ eyes (B). C, D) K12 expression was found in corneal epithelium of *WT* eyes but not in *Fgfr2* ^*CKO*^ eyes, suggesting that corneal epithelial cell differentiation was abnormal in mutant eyes. (LE = lens epithelium.)

At the stages between E16.5 to P0 (Fig. 1 E–H), the *WT* corneal epithelium consists of two cell layer, so as in the *Fgfr2* ^*CKO*^ cornea. However, the cell shape is changed from cuboidal to flattened in the mutant epithelium, suggesting differentiation could be affected by *Fgfr2* deletion. The hallmark of corneal epithelial differentiation and maturation is the expression of keratin 12 (K12) by E14.5 [35]. K12 is not expressed in the limbal and conjunctival epithelium. In contrast to K12, K14 is expressed in all ocular surface epithelial tissues. To investigate whether loss of FGFR2 in the corneal epithelium affects cell differentiation, K12 and K14 immunofluorescence was examined in E16.5 *WT* and *Fgfr2* ^*CKO*^ eyes. The results showed that although the corneal epithelial layer was significantly thinner in *Fgfr2* ^*CKO*^ eyes, K14 was expressed in this cell layer in *Fgfr2* ^*CKO*^ eyes, as well as in *WT* eyes (Fig. 5A and 5B). However, K12 expression was not detected in *Fgfr2* ^*CKO*^corneal epithelium but was in *WT* corneal epithelium (Fig. 5C and 5D). This result suggests that loss of FGFR2 affects corneal epithelial cell differentiation and maturation.

**Fig 6:**
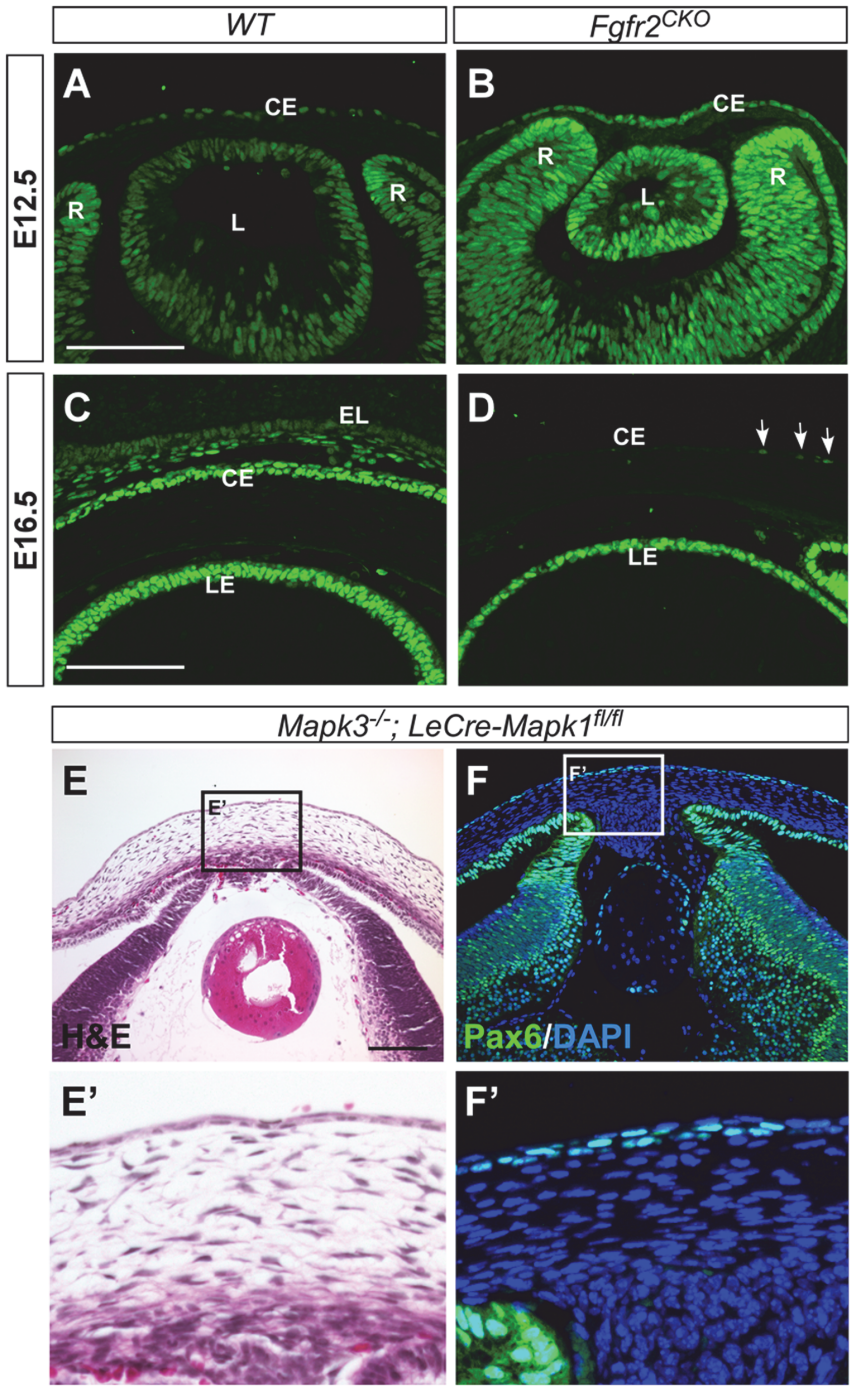
Pax6 immunofluorescence. A, B) In E12.5 *WT* and *Fgfr2* ^CKO^ eyes, Pax6 was expressed in developing corneal epithelial (CE), lens (L) and retinal (R) cells. The expression patterns were similar between *WT* and *Fgfr2* ^*CKO*^ eyes. C, D) At E16.5, Pax6 expression was found in corneal and conjunctival epithelium in *WT* eyes (C) but was significantly reduced in corneal epithelium of *Fgfr2* ^*CKO*^ eye, with a weak signal detected in a few cells (arrows in D). LE, lens epithelium. E, F) Deletion of *Mapk1* and *Mapk3*, encoding for ERK2 and ERK1 respectively, in the surface ectodermal-derived tissues severely affected lens and corneal development (H&E staining in E and E’). However, Pax6 expression appears to be normal in these tissues (F and F’).

Transcription factor Pax6 is known to be essential for K12 expression and corneal development. Pax6 is expressed in several ocular cell types, including the corneal and limbal epithelia, where gene expression is maintained throughout life [36–39]. In E12.5 *WT* and *Fgfr2* ^*CKO*^ eyes, Pax6 was expressed in developing corneal epithelium as well as in the lens and retina (6A and 6B). The expression patterns were similar in *WT* and *Fgfr2* ^*CKO*^ eyes. In E16.5 *WT* cornea, Pax6 expression was also found in conjunctival epithelium of the eyelid (Fig. 6C). In contrast, Pax6 immunofluorescence was almost abolished in *Fgfr2* ^*CKO*^ cornea and a low-level expression was detected in a few cells (labeled by arrows in Fig. 6D). This finding suggests that FGFR2 plays a critical role in maintaining Pax6 expression. Loss of Pax6 expression could result in the absence of K12 expression and account for the abnormal differentiation of corneal epithelium in *Fgfr2* ^*CKO*^ mice.

ERK is one of the major downstream effector of FGFR activation. To investigate whether FGFR2′s role of maintaining Pax6 level requires ERK activity, we examined the Pax6 expression in the E16.5 cornea of ERK1/2 double conditional deletion mice [29]. We found that, in contrast to the *Fgfr2* ^*CKO*^ cornea, Pax6 expression was maintained normally in the ERK1/2-deficient corneal epithelial cells (Fig. 6 F and F’), suggesting that FGFR2 controls the Pax6 level through an ERK-independent signaling pathway. In the ERK1/2-deficient cornea, the stromal layer looked abnormal and corneal endothelium was absent, probably due to the severe defective and degenerative lens in these mice [40].

### Abnormal differentiation of corneal mesenchymal cells in *Fgfr2* ^*CKO*^ mice

**Fig 7:**
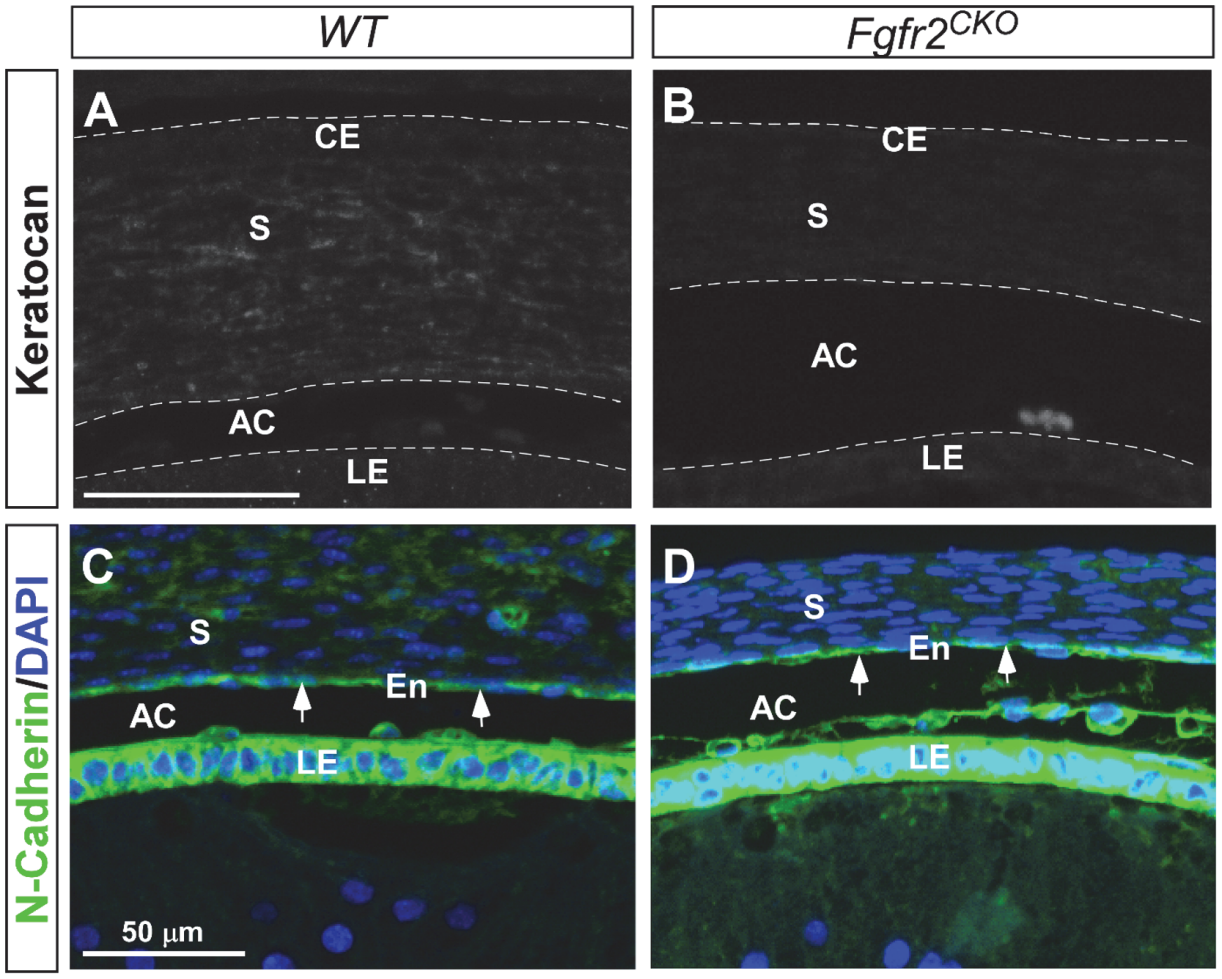
Corneal mesenchymal cell differentiation at E16.5 in *WT* and *Fgfr2* ^CKO^ eyes. A, B) Keratocan expression was found in the stroma (S) of *WT* cornea, indicating the formation of keratocytes. Keratocan was not detected in the stroma of *Fgfr2* ^*CKO*^ cornea, suggesting that normal differentiation of corneal mesenchymal cells into keratocytes was disrupted. C, D) N-cadherin was expressed in corneal endothelium (En, arrows) in both *WT* and *Fgfr* ^*CKO*^ corneas. N-cadherin was also highly expressed in the lens epithelium (LE) in both genotypes. (AC = anterior chamber.)

Cre expression in the *Le-Cre* mice is limited to the ocular surface ectodermal tissues [27,29], however, developmental defects were seen in the mesenchyme-derived tissues, such as the corneal stroma. We assessed whether differentiation of corneal mesenchymal cells was affected by defective corneal epithelium in *Fgfr2* ^*CKO*^ mice. Keratocan is a cornea-specific keratan sulfate proteoglycan, and is considered a phenotypic marker for keratocytes [41,42]. Keratocan expression was found in the stroma of *WT* eyes but not in *Fgfr2* ^*CKO*^ eyes (Fig. 7A and 7B). In contrast, expression of N-cadherin, a marker for corneal endothelial cells [43], was seen in eyes of both genotypes (Fig. 7C and 7D). Taken together, the data suggest that abnormal differentiation of the corneal epithelial cells affects the normal differentiation of corneal keratocytes but not endothelial cells in *Fgfr2* ^*CKO*^ mice.

**Fig 8:**
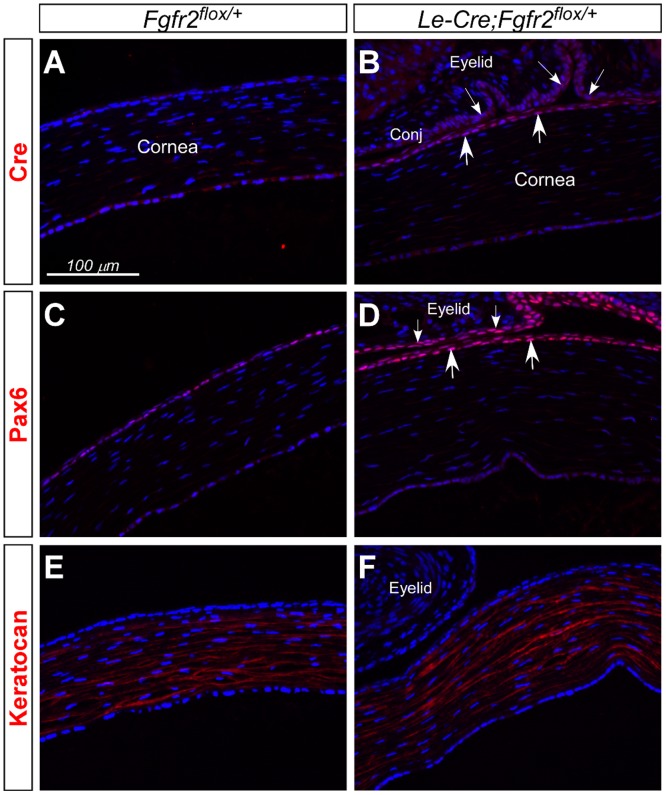
Cre, Pax6 and keratocan immunofluorescence in corneas of *Fgfr2* ^flox/+^ heterozygous mice with and without *Le-Cre* transgene at P5. A, B) Cre was expressed in corneal (arrows) and conjunctival (small arrows) epithelium of *Le-Cre;Fgfr2* ^*flox/+*^ eyes (B), but not in *Fgfr2* ^*flox/+*^ eyes (A). C-F) Pax6 expression was found in corneal (arrows, D) and conjunctival (small arrows, D) epithelium, and keratocan expression in corneal stroma (E, F). The expression patterns of these proteins were similar in corneas between *Fgfr2* ^*flox/+*^ (C, E) and *Le-Cre;Fgfr2* ^*flox/+*^ eyes (D, F). Note that histological artifact caused the corneas in *Fgfr2* ^*flox/+*^ eyes (without eyelids) to appear slightly thinner than those in *Le-Cre;Fgfr2* ^*flox/+*^ eyes (with eyelids).

Throughout the study, we have used *Fgfr2* ^*flox/flox*^ mice as a control (referred as *WT*) to compare with *Le-Cre;Fgfr2* ^*flox/flox*^ mice (or *Fgfr2* ^*CKO*^). Recently it was reported that hemizygous *Le-Cre* transgenic mice can develop severe eye defects on some genetic background [30]. We examined the expression of Pax6 and keratocan in the corneas of *Le-Cre;Fgfr2* ^*flox/+*^ heterozygous mice at postnatal day 5 (P5) (Fig. 8, C-F). We found no significant changes in the expression of these proteins in corneas between *Fgfr2* ^*flox/+*^ and *Le-Cre;Fgfr2* ^*flox/+*^ mice. This result suggests that the changes we have demonstrated in this study are unlikely caused by the expression of *Le-Cre* transgene in corneal epithelium.

## Discussion

### FGFR2 is required for corneal epithelial cell proliferation during early eye development

In early steps of eye development, FGF-7 and FGF-10 are expressed in the perioptic mesenchyme, some of these cells migrate into the space between lens and corneal epithelial layer to form the presumptive corneal stroma [20,44]. Both FGF-7 and FGF-10 are ligands for FGFR2. In the previous “gain-of-function” studies, overexpression of FGF-7 and FGF-10 driven by the lens-specific αA-crystallin promoter resulted in suppression of corneal epithelial cell fate and induction of ectopic lacrimal gland formation at the corneal surface of transgenic mice [20–22]. These results suggest that tight regulation of FGFR-signaling in corneal epithelium plays a critical role in controlling cell fate determination and commitment. In this study, we used a surface ectodermal Cre driver (*Le-Cre*) and demonstrate that loss of FGFR2 in corneal epithelial cells causes significant decrease in cell proliferation without affecting the epithelial cell fate and cell survival. Combining the ligand expression patterns with our data from this “loss-of-function” study, we propose that cell proliferation in the prospective corneal epithelium is regulated by FGFR2-signaling through epithelial-mesenchymal interaction in a paracrine fashion.

The mature corneal epithelium is a stratified tissue that continuously renews itself. The mitogens for later stage and older corneas are likely secreted by the lacrimal gland and distributed via the tears over the ocular surface. For example, epidermal growth factor (EGF) and transforming growth factor α (TGFα) exist as a component of human tears. EGF and TGF-α share a common EGF receptor (EGFR). Both growth factors are capable to stimulate corneal epithelial cell proliferation in vitro and in vivo [3,43,45]. Other growth factors, including hepatocyte growth factor (HGF), FGF-7, insulin-like growth factor (IGF)-1 and IGF-2 had all been proved to stimulate corneal epithelia cells proliferation in a dose-dependent manner in vitro [15,46–48]. HGF and FGF-7 are expressed in stromal keratocytes and are highly upregulated following epithelial injury [48]. The levels of FGF-7 and FGFR2 transcripts were highest in limbal fibroblasts and epithelial cells respectively in the periphery [49], while the expression of HGF and its receptor is higher in central cornea [14], suggesting a regional specificity of these two growth factor-signaling in control of cell proliferation. In our study, we also demonstrate that the cell proliferation indices in the peripheral corneal epithelium are higher than that in the central region in both *WT* and *Fgfr2* ^*CKO*^ (Fig. 4). The role of FGFR2 in later developmental stage and in mature cornea can be investigated using an inducible Cre line.

### Corneal epithelial cell fate is specified but cannot commit for further differentiation and maturation in *Fgfr2* ^*CKO*^ mice

We demonstrate that cytokeratin K12 (K12), a marker for corneal epithelial cell differentiation, is not expressed in *Fgfr2* ^*CKO*^ eyes (Fig. 6) [50], suggesting the FGFR2-signaling is also required for corneal epithelial cell differentiation and maturation. However, K14 is expressed in both *WT* and *Fgfr2* ^*CKO*^ corneal epithelium, suggesting that corneal epithelial cell fate is specified but remains in a less differentiated form. One potential mechanism for the differentiation defect in *Fgfr2* ^*CKO*^ mice might be due to the loss of Pax6 expression in corneal epithelium. We show that Pax6 is expressed in E12.5 corneal epithelial layer of *Fgfr2* ^*CKO*^ eyes but was almost absent at later stage (E16.5), suggesting that FGFR2-signaling activity plays an important role in maintaining Pax6 level in corneal epithelium. Similar observation was reported by Faber et al that, when a dominant negative FGFR1 is expressed in the lens, Pax6 expression levels were reduced [51]. However, the underlying mechanism of FGFR-signaling in maintaining Pax6 level might be different between these two surface ectodermal derived tissues. For example, FGFR-ERK signaling plays a major role in lens development, whereas ERK activity (judged by phosphorylated ERK level) was hardly detectable in the developing corneal epithelium (S1 Fig.) and loss of ERK1/2 did not affect the Pax6 levels in these cells (Fig. 6E–F’). The downstream signal transduction pathways of FGFR2 in corneal epithelial cells require further investigation.

Pax6 is known to be essential for normal corneal morphogenesis [52,53]. During development, Pax6 is expressed in the surface ectoderm prior to and during corneal epithelial differentiation, and is maintained in the adult corneal epithelium including the limbal region where the stem/progenitor cell population exists [38]. Pax6 functions as a co-activating factor for K12 expression [54]. K12 expression was reduced in Pax6^+/-^ mouse cornea [55]. Thus, loss of Pax6 in *Fgfr2* ^*CKO*^ cornea can directly affect activation of genes, such as K12, which are crucial for corneal epithelial cell differentiation and maturation. Additionally cellular adhesion was also compromised in the Pax6^+/-^ mutant corneal epithelium [56]. In postnatal and adult Pax6^+/-^ mouse cornea, the epithelial layer is thinner owing to a reduction in the number of cell layers, despite a tenfold increase in the proliferative index and no change in TUNEL labeling. Because proliferation of limbal and corneal epithelial cells in Pax6^+/-^ mice was not reduced, it confirms that decrease of cell proliferation in corneal epithelium of *Fgfr2* ^*CKO*^ mice is a direct result of FGFR2-deficiency not due to the loss of Pax6.

It is worthwhile to mention that during normal development FGFR-signaling level in corneal epithelial cells must be under tight control to ensure normal differentiation. As mentioned in the “Introduction”, FGF-3, FGF-7 and FGF-10 are the ligands of FGFR2. Overexpression of any of these FGFs from the lens at early developmental stage can alter the corneal epithelial cell fate, initially induce these cells to over-proliferate and then differentiate into secretory cell types (e.g. lacrimal and Harderian glands) [20–22]. Expressing an active form of Ras in corneal epithelium can also inhibit K12 expression [57]. The gain- and loss-of-function studies all indicate that proper corneal epithelium differentiation and maturation depend on the tightly controlled FGFR-signaling activity in these cells.

### Interaction between corneal epithelium and underlying mesenchyme is critical for cell fate commitment and differentiation in both tissues

When the lens vesicle and primitive corneal epithelium are completely separated, the space between them is filled by invading corneal mesenchymal cells which are mostly of neural crest origin. In mouse, the posterior mesenchyme cells closest to the lens differentiate into the corneal endothelium and subsequently the anterior chamber is formed. The mesenchyme cells between the corneal epithelium and endothelium start to differentiate into stromal keratocytes [2,58]. One of the differentiation markers for keratocytes is keratocan, a type of keratan sulfate-containing proteoglycans (KSPGs) uniquely abundant in the corneal stroma [41]. In *Fgfr2* ^*CKO*^ corneal stroma, keratocan was not expressed, even though the mesenchymal cell proliferation was not affected, suggesting that differentiation of keratocytes was disrupted by *Fgfr2* deletion in the corneal epithelium. While more molecular markers for keratocyte differentiation need to be examined, we suspect that the stromal cell differentiation defect could result from loss of Pax6 expression in the corneal epithelial layer. In *Pax6* ^*+/-*^ mutant embryos, corneal epithelium was abnormal in K12 expression and was thinner than *WT* littermates, and corneal stroma appeared irregular, hypercellular, and thickened [39,59]. However, the abnormalities seen in *Fgfr2* ^*CKO*^ stroma did not completely resemble the defects in *Pax6* ^*+/-*^ cornea, suggesting that in addition to lower level of Pax6, other factors as a result of FGFR2-signaling in corneal epithelium also contribute to stromal cell differentiation in normal corneal development.

### FGFR2 plays a different role in corneal epithelium and lens development

During vertebrate eye development, lens and corneal epithelium arise from the same surface ectoderm and share several common features, for example, they are both transparent and require sustained expression of Pax6. At E11.5–12.0, the lens vesicle detaches from the overlying surface ectoderm which subsequently forms the prospective corneal epithelium (Fig. 1A). Our study demonstrates that FGFR2 plays a different role in these two ectodermal tissues with the same origin. In the lens, FGFR2 is required for cell survival and cell cycle withdrawal during fiber differentiation, but it is dispensable for cell proliferation [25]. In contrast, FGFR2 is essential for cell proliferation in corneal epithelium but is dispensable for cell survival. The different response is likely caused by different downstream signal transduction pathways activated by FGFR2. Previous study by Burgess et al showed that activation of Ras, a downstream effector of FGF-signaling, triggers different sets of downstream targets in lens and in corneal epithelium [57]. Constitutive activation of Ras initially increased cell proliferation in both lens and corneal epithelial cells, correlating with increased levels of cyclin D1 and D2 expression in both cell types. This initial increase was sustained in the corneal epithelium, but not in the lens. Instead, cell cycle inhibitors, p27^kip1^ and p57^kip2^, were upregulated in the lens followed by hyperproliferation, probably through upregulation of transcription factor Prox1. Furthermore, the downstream effectors of FGFR2-Ras signaling also differ between lens and corneal epithelial cells. For example, the phospho-ERK1/2 level was increased in the lens, but not in the corneal epithelial cells, in response to Ras activation. When we examined the pERK1/2 levels in E12.5 cornea by immunofluorescence, we found that the pERK1/2 level was hardly detectable in central corneal epithelium in either *WT* or *Fgfr2* ^*CKO*^ eyes. Double deletion of *Mapk1* and *Mapk3* (encoding for ERK2 and ERK1 respectively) in the surface ectoderm did not cause any visible changes in early steps of central corneal development (Fig. 6, E-F’) although the cell numbers appeared to be reduced in the peripheral area near conjunctival fornix (data not shown), suggesting that role of FGFR2 in central corneal development is not controlled by ERK-signaling. This result is also consistent with the previous report in Ras-overexpression transgenic mice [57]. We conclude that FGFR2-signaling activates ERK-dependent and ERK-independent pathways in the lens and in central corneal epithelial cells respectively [29]. The signaling effectors and targets of FGFR2 in corneal epithelial cells have not been well defined and needs to be further investigated.

In summary, we demonstrate that FGFR2-activated ERK-independent signal is essential for corneal epithelial cell proliferation during early eye development. In later stage, FGFR2 is also critical for corneal epithelial cell differentiation and maturation, one potential mechanism is to maintain Pax6 expression in corneal epithelium. In contrast to the ocular lens, FGFR2 is dispensable for survival of corneal epithelial cells. Our study implies that keratocyte differentiation in corneal stroma depends on some inductive signals released from the overlying corneal epithelium. More studies are needed to investigate the downstream signaling events of FGFR2 in corneal epithelium and signals/factors from the corneal epithelial cells responsible for keratocyte differentiation.

## Supporting Information

S1 Fig Phospho-ERK (pERK) immunofluorescence in developing mouse eyes. Frozen sections of E12.5 eyes (A, B) and paraffin-sections of E14.5 (C, D) and P0 (E, F) eyes were used for immunostaining using an anti-pERK antibody from Cell Signaling Technology Inc. (Cat#9101). TSA enhancement reagents (Life Technology, Cat#T20948) were used on E14.5 eye sections (C, D) to increase the immunofluorescence signals. We found that pERK proteins were localized in elongating fiber cells of E12.5 lenses and in the cortical region of E14.5 and P0 lenses (indicated by arrowheads). pERK was also found in the retina of E12.5 and E14.5 eyes. pERK signal in cornea epithelium was not detected under standard immunostaining condition (A, B, E, F), however, a low level of pERK was detected in E14.5 corneal epithelium when TSA was used (arrows in C, D).
(TIF)
