# Supplementary figures and images for: Fibroblast Growth Factor Receptor 2 (FGFR2) Is Required for Corneal Epithelial Cell Proliferation and Differentiation during Embryonic Development
Source: PLoS One. 2015 Jan 23;10(1):e0117089. doi: 10.1371/journal.pone.0117089 (PMC4304804; doi:10.1371/journal.pone.0117089)

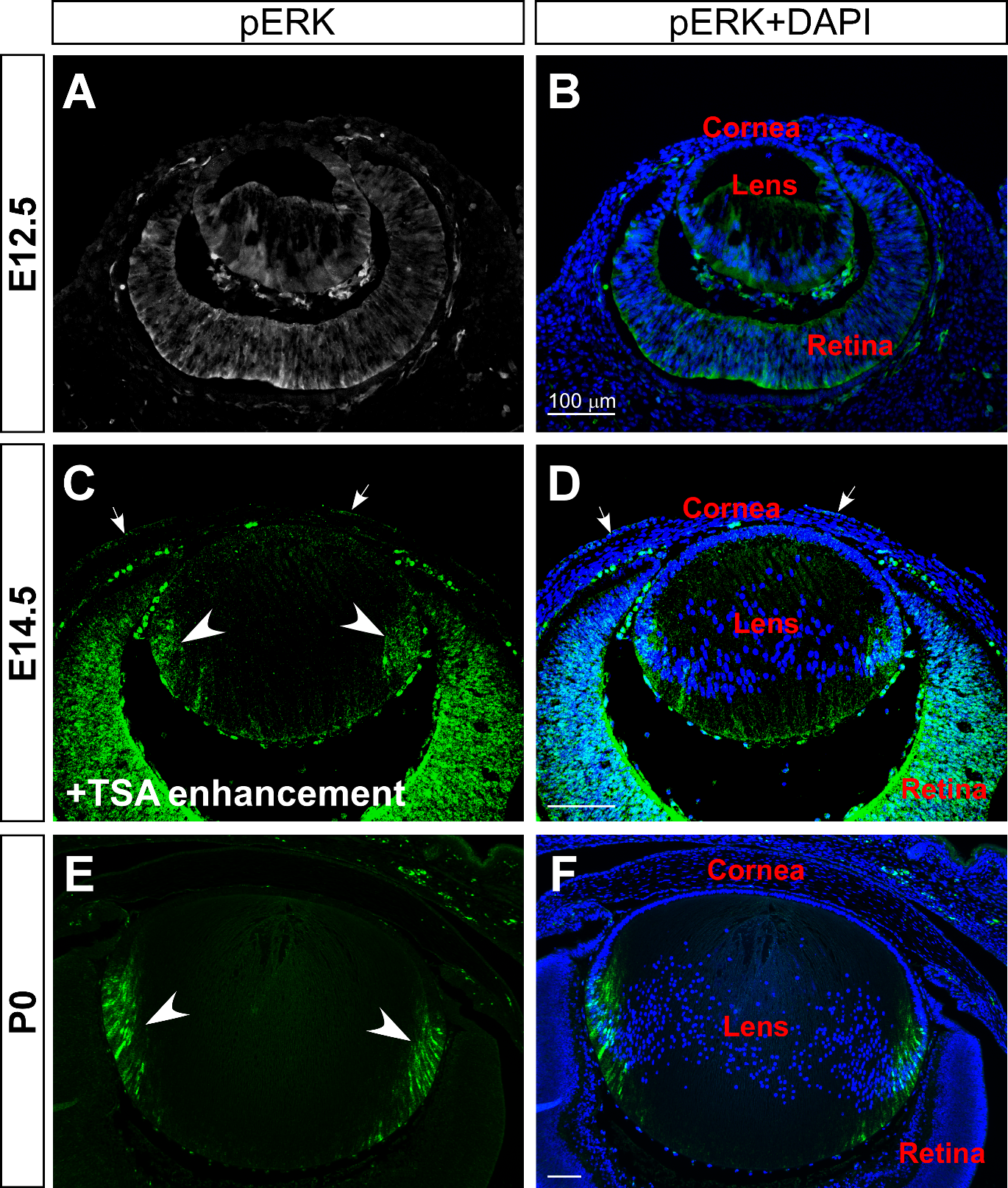

Supplement: S1 Fig — Frozen sections of E12.5 eyes (A, B) and paraffin-sections of E14.5 (C, D) and P0 (E, F) eyes were used for immunostaining using an anti-pERK antibody from Cell Signaling Technology Inc. (Cat#9101). TSA enhancement reagents (Life Technology, Cat#T20948) were used on E14.5 eye sections (C, D) to increase the immunofluorescence signals. We found that pERK proteins were localized in elongating fiber cells of E12.5 lenses and in the cortical region of E14.5 and P0 lenses (indicated by arrowheads). pERK was also found in the retina of E12.5 and E14.5 eyes. pERK signal in cornea epithelium was not detected under standard immunostaining condition (A, B, E, F), however, a low level of pERK was detected in E14.5 corneal epithelium when TSA was used (arrows in C, D). (TIF) [file pone.0117089.s001.tif]
